# Association between polymorphisms in the *GRIN1* gene 5′ regulatory region and schizophrenia in a northern Han Chinese population and haplotype effects on protein expression in vitro

**DOI:** 10.1186/s12881-019-0757-3

**Published:** 2019-01-31

**Authors:** Yong-ping Liu, Mei Ding, Xi-cen Zhang, Yi Liu, Jin-feng Xuan, Jia-xin Xing, Xi Xia, Jun Yao, Bao-jie Wang

**Affiliations:** School of Forensic Medicine, China Medical University, No. 77 Puhe Road, Shenyang, 110,122 Shenbei New District China

**Keywords:** *GRIN1*, schizophrenia, association, polymorphism

## Abstract

**Background:**

Schizophrenia is a severe neurodevelopmental disorder with a complex genetic and environmental etiology. Abnormal glutamate ionotropic N-methyl-D-aspartate receptor (NMDA) type subunit 1 (NR1) may be a potential cause of schizophrenia.

**Methods:**

We conducted a case-control study to investigate the association between the *GRIN1* gene, which encodes the NR1 subunit, and the risk of schizophrenia in a northern Chinese Han population using Sanger DNA sequencing. The dual luciferase reporter assay was used to detect the influence of two different haplotypes on *GRIN1* gene expression.

**Results:**

Seven SNPs (single nucleotide polymorphisms), including rs112421622 (− 2019 T/C), rs138961287 (− 1962--1961insT), rs117783907 (−1945G/T), rs181682830 (−1934G/A), rs7032504 (-1742C/T), rs144123109 (−1140G/A), and rs11146020 (−855G/C) were detected in the study population. Rs117783907 (−1945G/T) was associated with the occurrence of schizophrenia as a protective factor. The genotype frequencies of rs138961287 (− 1962--1961insT) and rs11146020 (−855G/C) were statistically different between cases and controls (*p* < 0.0083). The other four variations were not shown to be associated with the disease. Two haplotypes were composed of the seven SNPs, and distribution of T-del-G-G-C-G-G was significantly different between the case and control groups. However, the dual luciferase reporter assay showed that neither of the haplotypes affected luciferase expression in HEK-293 and SK-N-SH cell lines.

**Conclusions:**

The *GRIN1* gene may be related to the occurrence of schizophrenia. Additional research will be needed to fully ascertain the role of *GRIN1* in the etiology of schizophrenia.

**Electronic supplementary material:**

The online version of this article (10.1186/s12881-019-0757-3) contains supplementary material, which is available to authorized users.

## Background

Schizophrenia is a serious genetic disease characterized by emotional impairment, cognitive deficits, and social dysfunction [1]. A large schizophrenia genome-wide association study (GWAS) recently indicated that multiple genes involved in glutamatergic neurotransmission were relevant to schizophrenia [2]. Glutamate is the primary excitatory neurotransmitter in the central nervous system (CNS) and exerts a physiological role by binding to several glutamate receptors. Of the various glutamate receptors, NMDA receptors have received more attention due to their pivotal role in axonal formation, long-term potentiation, and excitotoxicity [3, 4]. Studies have shown that NMDA receptors are closely related to learning and memory [5]. Moreover, a review [6] pointed out that abnormal NMDA receptors are associated with Alzheimer’s disease, Huntington’s disease, and epilepsy. Phenylcyclohexyl piperidine (PCP), which blocks a NMDA glutamate receptor subtype [7], induces a psychotomimetic state that closely resembles schizophrenia [8]. These findings imply that NMDA receptor dysfunction might be involved in the etiology of the CNS disorders [9].

NMDA receptors are heterotetramers composed of two NR1 subunits and NR2 or/and NR3 subunits [10]. The NR1 subunit, which is encoded by the *GRIN1* gene, is a functional subunit of the NMDA receptor and is widely distributed throughout the brain [11]. Mice that expressed only 5% normal levels of the NR1 subunit showed increased activity, dullness, and social and sexual deficiencies. Moreover, these behavioral changes were similar to those observed in animal models of schizophrenia [12]. mRNA and protein levels of NR1 subunits were shown to be decreased in the postmortem brain of schizophrenic patients [13, 14]. These studies indicate a potential association between the *GRIN1* gene and schizophrenia. Although one study [15] identified 143 differentially expressed proteins in the anterior cingulate cortex between schizophrenia patients and healthy controls, it did not include the NR1 subunit. In addition, genetic association studies showed no significant difference in genotypic and allelic frequency distribution of the *GRIN1* gene between schizophrenic and healthy controls in Japanese and Chinese populations [16, 17].

The role of *GRIN1* in the etiology of schizophrenia remains uncertain, and genetic association studies of the *GRIN1* gene and schizophrenia in the northern Chinese Han population are relatively deficient. We conducted a case-control study to investigate the association between *GRIN1* and the risk of schizophrenia in a northern Chinese Han population using Sanger DNA sequencing. Furthermore, the effects of two different haplotypes located in the 5′ promoter region of the *GRIN1* gene on protein expression were detected by dual luciferase reporter assay.

## Methods

### Samples

Blood samples from 316 northern Han Chinese healthy unrelated volunteers (157 females, 159 males, mean age 44 ± 14.3) were provided by China Medical University. Questionnaires showed that there was no history of mental illness within three generations. Blood samples from 309 northern Han Chinese patients with schizophrenia (156 females, 153 males, average age 41 ± 14.6) were provided by the Third People’s Hospital of Liaoning Province. The diagnosis of schizophrenia was in accordance with *The Diagnostic and Statistical Manual of Mental Disorders (fourth edition).* To confirm the diagnoses, two independent senior psychiatrists reviewed psychiatric medical records. Genomic DNA was extracted from peripheral blood by the standard phenol-chloroform method. The study was approved by the Ethics Committee of China Medical University, and written informed consent was obtained from each participant and/or patient guarantor.

### PCR amplification

Polymerase chain reaction (PCR) was used to amplify the *GRIN1* fragment, including the 5′ flanking and untranslated regions. The nucleotide position of the target fragment amplified was from − 2334 to + 86 (with ATG + 1). Genomic DNA (1 μL, about 30 ng) was amplified under the following reaction contents: 1 μL (5 pmol) each of sense and antisense primers, 2 μL (3 nmol) of dNTP mix, 0.2 μL (about 0.5 U) of PrimeSTAR® HS DNA polymerase (Takara, Dalian, China) and 10 μL 2 × Prime STAR HS GC buffer. Sterilized deionized water was added to a volume of 20 μL. PCR cycling conditions were 94 °C for 1 min; 30 cycles at 98 °C for 10 s, at 60 °C for 5 s, and 72 °C for 2 min 30 s; and 72 °C for 10 min. PCR products were separated by 1% agarose gel electrophoresis.

### DNA sequencing

DNA was sequenced using Sanger DNA sequencing (Taihe Biotechnology Co. Ltd. Beijing China). Primer information was shown in Table 1.

**Table 1:** Primers used for *GRIN1* gene sequencing

Primer name	Annealing temperature (°C)	Primer sequence (5′ → 3′)
F (−2334 — -2313)	60	5’ AGCTTGGGGACGCACATACGGT 3’
R1 (+ 64 — + 86)	60	5’ AATGTTGACGATCTTGGGGTCGC 3’
R2 (− 700 — -676)	–	5’ GATCACCTGCCCGTACCCTGCTGCA 3’
R3 (− 1515 — -1491)	–	5’ GTCGTCACCCACAGTCAGCGATATT 3’

F indicates the forward primer and R indicates the reverse primer. F and R1 are PCR amplification primers, and F, R1, R2, and R3 are Sanger sequencing primers. The position of the primer is in parentheses (with ATG + 1).

### Construction of pGL-3 recombinant vector

The target fragment located at − 2143 - + 222 (with ATG + 1) was amplified using a PrimerSTAR® kit (Takara, Dalian, China). The sense primer was 5’ GGCTAGCCTGAACATTTAGCGATCA 3′ and the antisense primer was 5’ CAGATCTGGCATTGAGCTGAATCTTC 3′. The primers contained *Nhe*I or *Bgl*II restriction endonuclease sites at the 5′ end. By using the pGM-T Ligation® Kit (TIANGEN, Beijing, China), PCR products purified from agarose gel were cloned into pGM-T vectors, and the recombinant vectors were subsequently transformed into JM109 competent cells. The pGM-T recombinant plasmids extracted by SanPrep® Column Enodotoxin-Free Plasmid Mini-Preps Kit (Sangon Biotech, Shanghai, China) were subjected to Sanger DNA sequencing to ensure correct insertion of the target fragment.

The two different haplotype pGM-T recombinant vectors were subcloned into the pGL-3 Basic Vector (Promega, Madison, Wisconsin, USA) using *Nhe*I or *Bgl*II.

### Cell culture

Human embryonic kidney cell line HEK-293 and neuroblastoma cell line SK-N-SH were used to test luciferase activity of the pGL-3 recombinant vectors. HEK-293 cells were cultured in HyClone® DMEM high glucose medium with 10% fetal bovine serum (Thermo Fisher Scientific, Massachusetts, USA). SK-N-SH cells were cultured in KeyGRN BioTECH® DMEM high glucose medium (with 0.011 g/L sodium pyruvate) with 15% fetal bovine serum. Cells were seeded in 24-well plates (2 × 10^5^ cells per well). According to the manufacturer’s protocol (Invitrogen, California, USA), Lipofectamine®3000 reagent was used to co-transfect the pGL-3 recombinant plasmids containing the two haplotypes with the Renilla luciferase-expressing vector pRL-TK (Promega) into the two cell lines. Cells were harvested after 24 h in culture. Firefly luciferase activity (LUC value) was measured and normalized to renilla luciferase activity (TK value). Each assay was performed in triplicate in the two cell lines.

### Statistical analysis

SPSS 20.0 software (IBM, Armonk, NY, USA) was used to analyze genotype frequency, allele frequency, haplotype frequency, and LUC/TK values (relative fluorescence intensity). Haploview 4.2 software (Broad Institute, Cambridge, MA, USA) was used for the Hardy–Weinberg equilibrium test and to confirm haplotypes. The χ2 test was used to examine the distribution of genotypes, alleles, and haplotypes between groups. The threshold for polymorphism multiple correction was *p* = 0.05/6, and the haplotype multiple test threshold after Bonferroni correction was 0.05/2. Independent sample T test was used to compare the relative fluorescence intensity of the two haplotypes. Relative fluorescence intensity was expressed as the mean ± standard deviation, and *p* < 0.05 (two sided) represented a significant difference. Power analysis was conducted using PS program [18] statistical software. Detection of *GRIN1* gene expression in different tissues was performed with the GTEx database (https://gtexportal.org/home/).

## Results

**Fig. 1:**
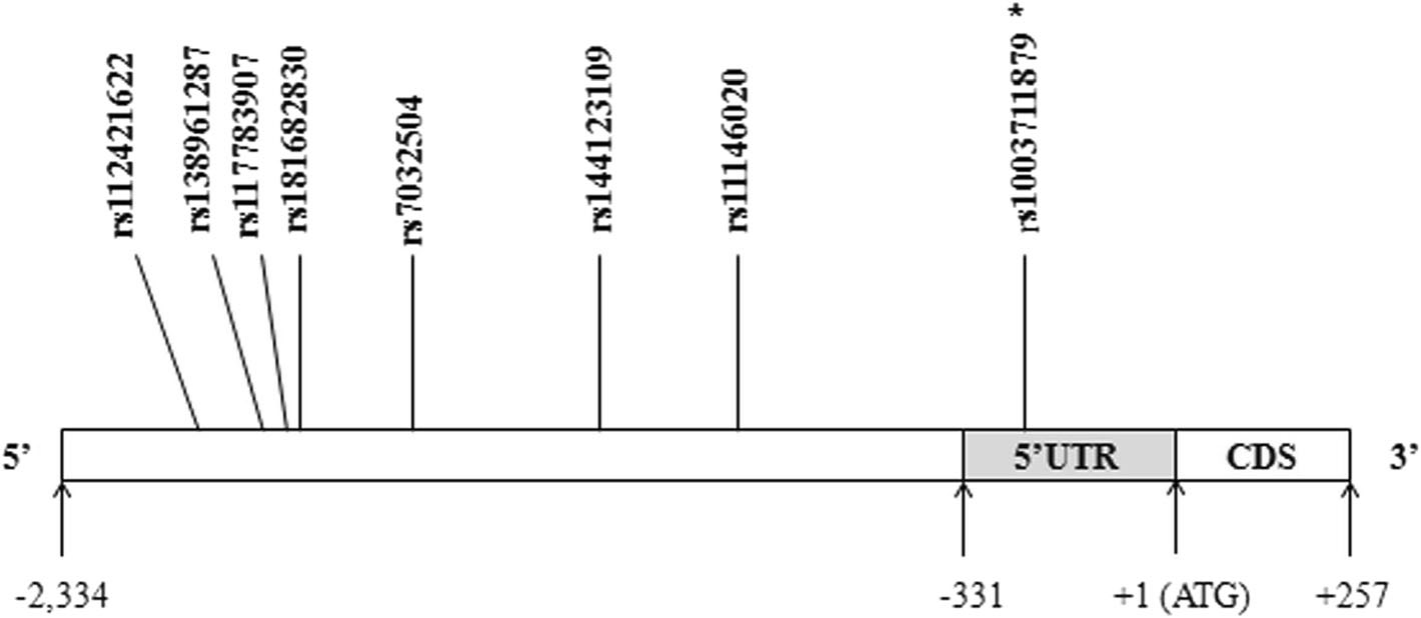
Schematic diagram of the distribution of seven SNP loci in the *GRIN1* gene. The detailed position of the fragment used for Sanger DNA sequencing in the *GRIN1* gene 5′ promoter region was located between − 2334 bp and + 86 bp. * denotes the SNP not included in the genetic association study because the minimum allele frequency was less than 0.01

**Fig. 2:**
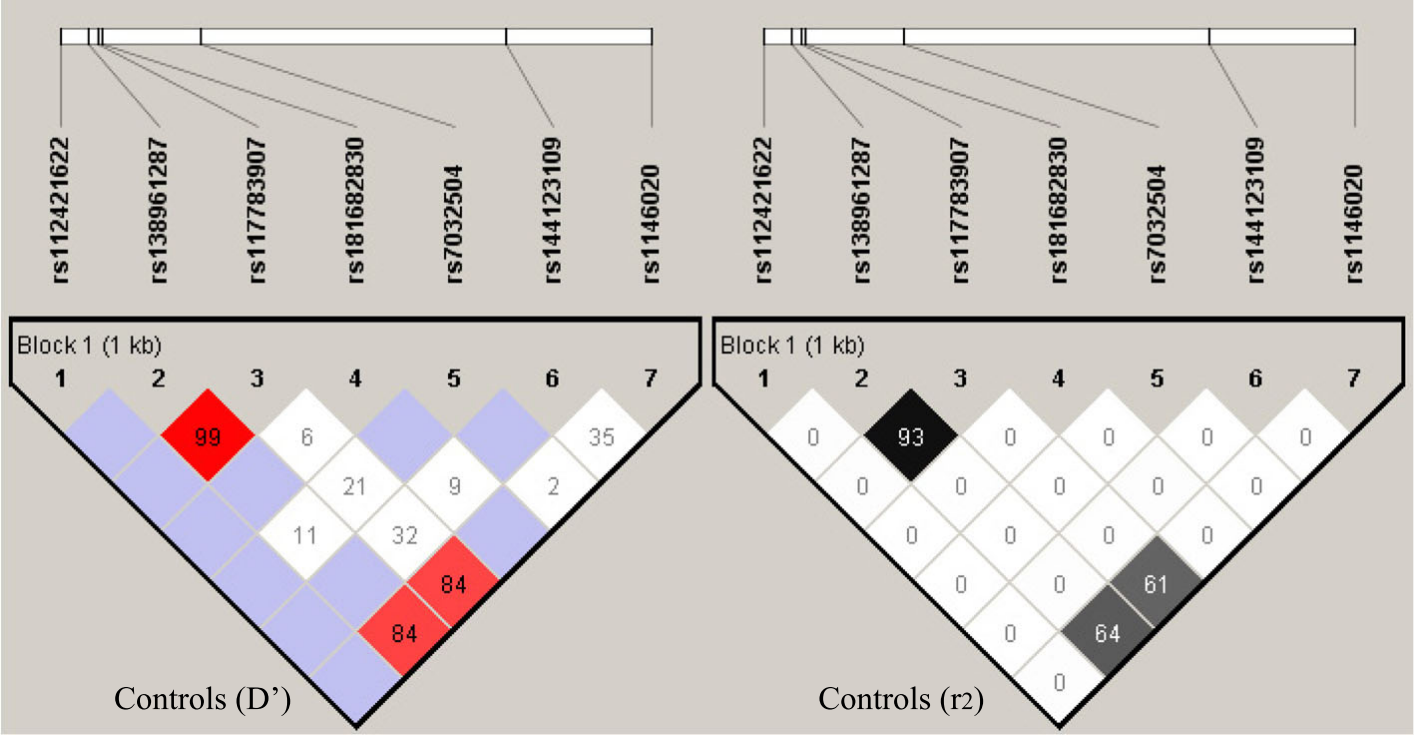
Linkage disequilibrium diagram of seven SNP loci in the *GRIN1* gene. Left is D’ in the control group, and the red grid represents D’ = 1. Right is r^2^ in the control group, and the black grid represents r^2^ = 1

Seven common SNPs (Fig. 1), including rs112421622 (− 2019 T/C), rs138961287 (− 1962--1961insT), rs117783907 (−1945G/T), rs181682830 (−1934G/A), rs7032504 (-1742C/T), rs144123109 (−1140G/A) and rs11146020 (−855G/C), were detected in the 5′ promoter region of the *GRIN1* gene in healthy Chinese Han individuals. The distribution of all seven SNPs was in accordance with Hardy-Weinberg equilibrium in the control group (*p* > 0.05). Results of linkage disequilibrium analysis using Haploview 4.2 software are presented in Fig. 2, showing that rs138961287 (− 1962 - -1961insT) and rs117783907 (−1945G/T) are in linkage disequilibrium (D’ = 0.99, r2 = 0.94). Given that there were six independent SNPs, the threshold for polymorphism multiple correction was *p* = 0.05/6 = 0.0083.

**Fig. 3:**
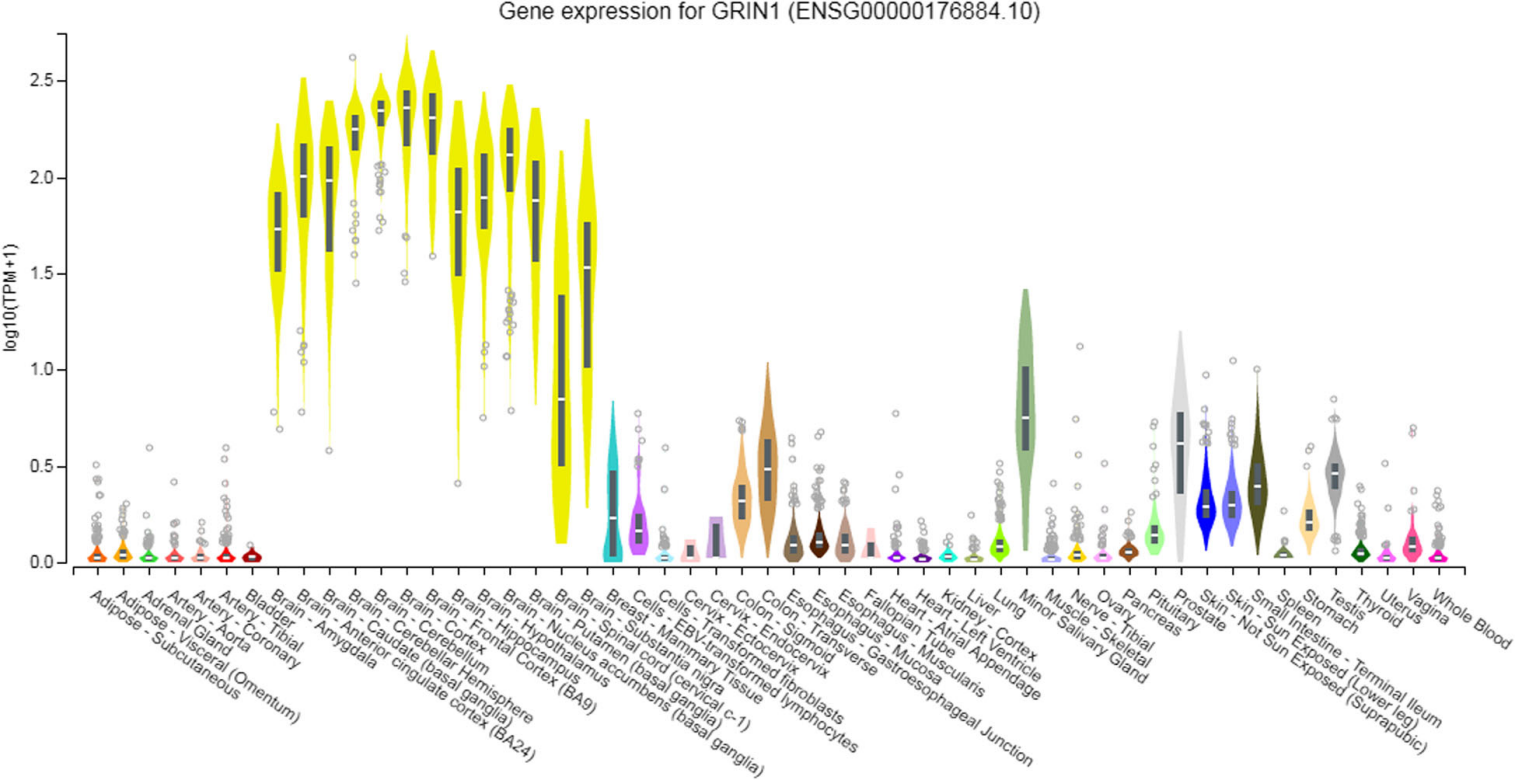
*GRIN1* gene expression in different tissues. TPM on the vertical axis represents the transcript quantification value, and the horizontal axis represents different tissues. The *GRIN1* gene was highly expressed in brain tissue compared with other tissues

Both genotype and allele distribution (Additional file 1) of rs117783907 (−1945G/T) were significantly different between the case and control groups (*p* < 0.0083). The frequency of the T allele in rs117783907 (−1945G/T) in the case group (15.2%) was much lower than in the control group (21.7%). The genotype frequencies of rs138961287 (− 1962--1961insT) and rs11146020 (−855G/C) were statistically different between cases and controls (p < 0.0083). Rs112421622 (− 2019 T/C), rs181682830 (−1934G/A), rs7032504 (-1742C/T), and rs144123109 (−1140G/A) were detected only in two genotypes and were determined to be unrelated to schizophrenia. The power of (− 1962 - -1961insT), rs117783907 (−1945G/T), and rs11146020 (−855G/C) were all > 0.70, and the power of rs117783907 (−1945G/T) in particular reached 0.982 (Additional file 1). According to the GTEx database, the *GRIN1* gene is highly expressed in brain tissue compared with other tissues (Fig. 3).

A total of seven SNPs formed six haplotypes, but after excluding four with very low frequencies (< 5%), only haplotypes T-del-G-G-C-G-G and T-insT-T-G-C-G-C remained. Therefore, the haplotype multiple test threshold after Bonferroni correction was 0.05/2 = 0.025. We found a significant difference in the haplotype frequency distribution of T-del-G-G-C-G-G between case and control groups (Additional file 2), with *p* values of 0.005. Specifically, the frequency of haplotype T-del-G-G-C-G-G in the case group (77.8%) was much higher than in the control group (70.9%), increasing the disease risk (OR = 1.442, 95% CI: 1.116–1.862). Haplotype T-insT-T-G-C-G-C was not related to schizophrenia.

**Fig. 4:**
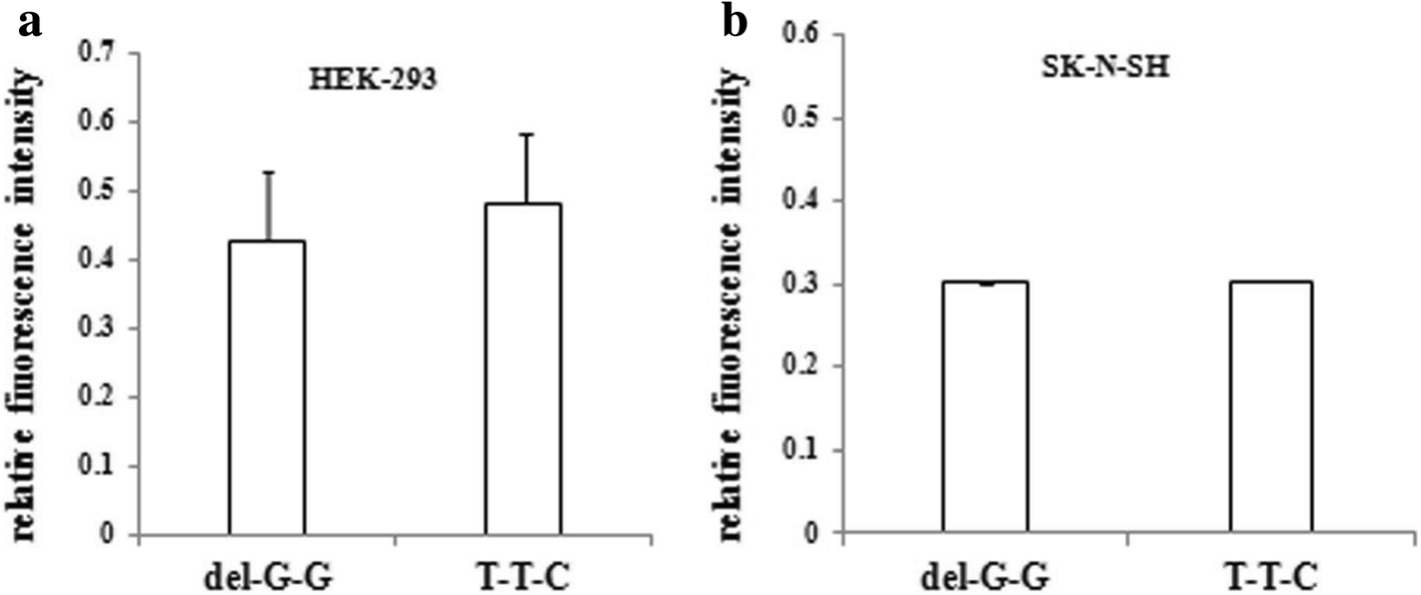
Relative fluorescence intensity of two different recombinant haplotypes in HEK-293 and SK-N-SH cells. **a** and **b** represent the relative fluorescence intensity (LUC/TK values) of two haplotypes in HEK-293 cells and SK-N-SH cells, respectively. Del-G-G represents the haplotype T-del-G-G-C-G-G; T-T-C represents the haplotype T-insT-T-G-C-C. There was no significant difference in relative fluorescence intensities between the T-del-G-G-C-G-G and T-insT-T-G-C-G-C haplotypes in HEK-293 (*p* = 0.178) or SK-N-SH cells (*p* = 0.959)

Comparing the LUC/TK values of the two haplotypes (Fig. 4), we found that the relative fluorescence intensity of the recombinant vector T-del-G-G-C-G-G was higher than T-T-T-G-C-G-C in HEK-293 cells. However, the difference was not statistically significant (*p* = 0.178). In SK-N-SH cells, no statistical difference in relative fluorescence intensity was found between the recombinant vectors T-del-G-G-C-G-G and T-T-T-G-C-G-C (*p* = 0.956).

## Discussion

In the present study, we examined the association between the *GRIN1* gene and the risk of schizophrenia in a northern Chinese Han population. Using Sanger DNA sequencing, we detected seven SNPs, including rs112421622 (− 2019 T/C), rs138961287 (− 1962--1961insT), rs117783907 (−1945G/T), rs181682830 (−1934G/A), rs7032504 (-1742C/T), rs144123109 (−1140G/A) and rs11146020 (−855G/C). Novel mutations were not detected.

Both genotype and allele distribution of rs117783907 (−1945G/T) were significantly different between the case and control groups (*p* < 0.0083). The genotype frequencies of rs138961287 (− 1962--1961insT) and rs11146020 (−855G/C) were statistically different between the case and control groups (*p* < 0.05), indicating that rs138961287 (− 1962--1961insT), rs117783907 (−1945G/T) and rs11146020 (−855G/C) are related to schizophrenia. Another association study in a northern Chinese Han population reported that the C allele frequency of rs11146020 reduced the risk of schizophrenia [19], although this allele was reported to be associated with schizophrenia as a risk factor in an Italian population [20]. Furthermore, a meta-analysis [21] suggested that the C variation allele of rs11146020 might be associated with an increased risk for developing schizophrenia.

The risk of developing psychosis increases with the accumulation of many genetic risk variants and exposure to multiple adverse environmental factors [22]. In different ethnic groups, the effects of environmental and genetic factors and their interactions on the risk of disorders may vary [21]. It has been shown that some genetic polymorphisms might be associated with altered risk in schizophrenia [23] and immune system diseases among different ethnicities [24–26]. Interestingly, recent evidence suggests that patients with schizophrenia might display signs of typical autoimmune processes associated with impaired functioning of microRNAs [27]. Thus, the opposite effect of this same C allele of rs11146020 (−855G/C) on schizophrenia in different ethnic groups is likely due to genetic heterogeneity.

There have been no studies involving the two SNPs in linkage disequilibrium rs138961287 (− 1962--1961insT) and rs117783907 (−1945G/T). The frequency of the T allele of rs117783907 (−1945G/T) in the case group was significantly lower than in the control group, indicating that it could be a protective factor in individuals of northern Han Chinese descent with schizophrenia.

According to the GTEx database, the *GRIN1* gene was highly expressed in brain tissues compared to other tissues, suggesting that *GRIN1* may play an important role in the CNS. Pranita et al. [28] found no significant risk for schizophrenia at the rs11146020 C allele. However, schizophrenic individuals in that study were being treated with antipsychotic drug therapy. Rice et al. [29] reported no association between the *GRIN1* gene and schizophrenia. However, the sample size was relatively small, with only five cases in an Asian population. Georgi et al. [30] detected distribution of four single nucleotide polymorphisms and one microsatellite marker at *GRIN1*. They found significant associations between schizophrenia and these polymorphisms in single-marker and haplotype-based analyses. Chanasong et al. [31] found that the A allele of rs1126442 was associated with increased risk of METH-dependent psychosis in a Thai population. Leuba et al. [32] indicated that patients with Alzheimer’s disease had significantly lower NR1 subunit levels in the entorhinal cortex and the frontal cortex. Wu et al. [33] genotyped six polymorphisms of *GRIN1* and *GRIN2B* and found that the two genes in conjunction with each other were associated with Parkinson’s disease in a Chinese population.

We defined two haplotypes (frequencies > 0.5) in the 5′ promoter region of the *GRIN1* gene. Haplotype T-del-G-G-C-G-G was statistically associated with increased risk of schizophrenia. Transcription factors regulate gene expression by interacting with cis-regulatory elements. Rice et al. [29] and Begni et al. [20] found that rs11146020 (−855G/C) may change the + 1 G of the transcription factor NF-κB consensus sequence (GGGG). However, we failed to detect significantly different luciferase expression between the two haplotypes in both the HEK-293 and SK-N-SH cell lines. In fact, the non-significant effects of the two haplotypes on luciferase expression is not inconsistent with the conclusion that the *GRIN1* gene is associated with schizophrenia. Because more than one transcription factor binds to cis-regulatory elements in the 5′ promoter region of the *GRIN1* gene, they likely reinforce or offset each other. A genome-wide association study [2] recently showed that multiple genes involved in the glutamatergic system and the *DRD2* gene in the dopamine system acted on schizophrenia, implying that the two systems do not work independently in schizophrenia. Similarly, it is reasonable to speculate that *GRIN1* and other interactional genes likely affect the function of *GRIN1* polymorphisms. Further studies are needed to verify this postulation.

There were several limitations in this study. First, in addition to the seven SNPs, another polymorphism (Fig. 1), rs1003711879 (− 289--287delGCC), was also detected. Unfortunately, due to the relatively small sample size, rs1003711879 (− 289 - -287delGCC) could not be included in the genetic association study because the frequency did not reach 1%. Second, only HEK-293 and SK-N-SH cell lines were used for functional experiments, and use of additional related cell lines may provide more information.

## Conclusions

Using Sanger DNA sequencing, we illustrated that rs11146020, rs138961287, and rs117783907 in the promoter region of the *GRIN1* gene are associated with schizophrenia in a northern Chinese Han population. Haplotype T-del-G-G-C-G-G might significantly increase the risk of schizophrenia, while T-insT-T-G-C-G-C is likely not related to disease risk. However, the dual luciferase reporter assay showed that neither of the haplotypes affected luciferase expression. Future efforts will be needed to ascertain the role of *GRIN1* in the etiology of schizophrenia.

## Additional files

Additional file 1: Genotype and allele distribution of SNPs in the *GRIN1* gene (significance threshold = 0.0083). ^a^ Frequency is shown in brackets (%); ^b^ *P* value shown in bold reaches a significant level (*P* < 0.0083); the genotype and allele frequency distribution of -1945G/T and the genotype frequency of -855G/C and − 1962 - -1961insT were significantly different between the case and control groups. (DOC 77 kb)

Additional file 2: Haplotypes formed by the seven SNPs in *GRIN1* and disease risk (significance level = 0.025). Del-G-G represents the haplotype T-del-G-G-C-G-G, T-T-C represents the haplotype T-insT-T-G-C-C.The frequency distribution of haplotype T-del-G-G-C-G-G was significantly different between the case group and the control group, with *p* values of 0.005. Specifically, the frequency of haplotype T-del-G-G-C-G-G in the case group (77.8%) was much higher than in the control group (70.9%), increasing the disease risk (OR = 1.442, 95% CI: 1.116–1.862). Haplotype T-insT-T-G-C-G-C was not related to schizophrenia (XLSX 12 kb)

## Abbreviations

95% CI: 95% confidence interval
ATG: transcription start site
CDS: coding sequence
OR: odds ratio
SNP: single nucleotide polymorphism
