# Supplementary material for: Association between polymorphisms in the GRIN1 gene 5′ regulatory region and schizophrenia in a northern Han Chinese population and haplotype effects on protein expression in vitro
Source: BMC Med Genet. 2019 Jan 31;20:26. doi: 10.1186/s12881-019-0757-3 (PMC6357472; doi:10.1186/s12881-019-0757-3)
Supplement: Supplementary file 1 — Genotype and allele distribution of SNPs in the GRIN1 gene (significance threshold = 0.0083). a Frequency is shown in brackets (%); b P value shown in bold reaches a significant level (P < 0.0083); the genotype and allele frequency distribution of -1945G/T and the genotype frequency of -855G/C and − 1962 - -1961insT were significantly different between the case and control groups. (DOC 77 kb) [file 12881_2019_757_MOESM1_ESM.doc]

| **Additional File 1. Genotype and allele distribution of SNPs in the *GRIN1* gene (significance threshold = 0.0083).** | | | | | | | | | | | | | |
| --- | --- | --- | --- | --- | --- | --- | --- | --- | --- | --- | --- | --- | --- |
| **SNP** | **Genotype^a^** | | | | ***P*-value^b^** | | **Allele^a^** | | ***P*-value^b^** | | **OR** | **95％CI** | **Power** |
| **rs112421622** | |  |  |  |  |  | |  |  |  | |  |  |
| **(-2019T/C)** | | TT | TC | CC |  | T | | C |  |  | |  |  |
| Case group | | 302 (97.7) | 7 (2.3) | 0 (0.0) | 0.265 | 611 (98.9) | | 7 (1.1) | 0.268 | 0.592 | | 0.231-1.514 | 0.197 |
| control group | | 304 (96.2) | 12 (3.8) | 0 (0.0) |  | 620 (98.1) | | 12 (1.9) |  |  | |  |  |
| **rs138961287** | |  |  |  |  |  | |  |  |  | |  |  |
| **(-1962-1961insT)** | | deldel | delinsT | insTinsT |  | del | | insT |  |  | |  |  |
| Case group | | 217 (70.2) | 90 (29.1) | 2 (0.7) | **0.001** | 524 (84.8) | | 94 (15.2) | 0.009 | 0.680 | | 0.508-0.909 | 0.741 |
| control group | | 202 (63.9) | 96 (30.4) | 18 (5.7) |  | 500 (79.1) | | 132 (20.9) |  |  | |  |  |
| **rs117783907** | |  |  |  |  |  | |  |  |  | |  |  |
| **(-1945G/T)** | | GG | GT | TT |  | G | | T |  |  | |  |  |
| Case group | | 217 (70.2) | 90 (29.1) | 2 (0.7) | **0.001** | 524 (84.8) | | 94 (15.2) | **0.003** | 0.648 | | 0.485-0.866 | 0.982 |
| control group | | 198 (62.7) | 99 (31.3) | 19 (6.0) |  | 495 (78.3) | | 137 (21.7) |  |  | |  |  |
| **rs181682830** | |  |  |  |  |  | |  |  |  | |  |  |
| **(-1934G/A)** | | GG | GA | AA |  | G | | A |  |  | |  |  |
| Case group | | 304 (98.4) | 5 (1.6) | 0 (0.0) | 0.424 | 613 (99.2) | | 5 (0.8) | 0.426 | 0.636 | | 0.207-1.956 | 0.126 |
| control group | | 308 (97.5) | 8 (2.5) | 0 (0.0) |  | 624 (98.7) | | 8 (0.3) |  |  | |  |  |
| **rs7032504** | |  |  |  |  |  | |  |  |  | |  |  |
| **(-1742C/T)** | | CC | CT | TT |  | C | | T |  |  | |  |  |
| Case group | | 301 (97.4) | 8 (2.6) | 0 (0.0) | 0.378 | 610 (98.7) | | 8 (1.3) | 0.380 | 1.645 | | 0.535-5.055 | 0.143 |
| control group | | 311 (98.4) | 5 (1.6) | 0 (0.0) |  | 627 (99.2) | | 5 (0.8) |  |  | |  |  |
| **rs144123109** | |  |  |  |  |  | |  |  |  | |  |  |
| **(-1140G/A)** | | GG | GA | AA |  | G | | A |  |  | |  |  |
| Case group | | 291 (94.2) | 18 (5.8) | 0 (0.0) | 0.236 | 600 (97.1) | | 18 (2.9) | 0.242 | 1.550 | | 0.740-3.245 | 0.216 |
| control group | | 304 (96.2) | 12 (3.8) | 0 (0.0) |  | 620 (98.1) | | 12 (1.9) |  |  | |  |  |
| **rs11146020** | |  |  |  |  |  | |  |  |  | |  |  |
| **(-855G/C)** | | GG | GC | CC |  | G | | C |  |  | |  |  |
| Case group | | 221 (71.5) | 85 (27.5) | 3 (1.0) | **0.004** | 527 (85.3) | | 91 (14.7) | 0.012 | 0.687 | | 0.511-0.923 | 0.706 |
| control group | | 207 (65.5) | 91 (28.8) | 18 (5.7) |  | 505 (79.9) | | 127 (20.1) |  |  | |  |  |
| Legend: ^a^ Frequency is shown in brackets (%); ^b^ *P* value shown in bold reaches a significant level (P<0.0083); the genotype and allele frequency distribution of -1945G/T and the genotype frequency of -855G/C and -1962 - -1961insT were significantly different between the case and control groups. | | | | | | | | | | | | | |
